# Medullary thyroid cancer in MEN2 pediatric/adolescent carriers of RET mutation: genotype/phenotype correlation and outcome in a retrospective series of 23 patients

**DOI:** 10.3389/fonc.2024.1464890

**Published:** 2025-01-07

**Authors:** Guenda Di Benedetto, Ignazio Barca, Laura De Gregorio, Claudia Scollo, Fiorenza Gianì, Federica Martorana, Marco Russo, Francesco Frasca, Gabriella Pellegriti, Giulia Sapuppo

**Affiliations:** ^1^ Endocrinology Unit, Garibaldi-Nesima Hospital, Department of Clinical and Experimental Medicine, University of Catania, Catania, Italy; ^2^ Molecular Biology Service, Multi-diagnostic Health Services Centre, Catania, Italy; ^3^ Endocrinology Service, Department of Internal Medicine, Maggiore Hospital, Modica, Italy; ^4^ Medical Oncology, Department of Clinical and Experimental Medicine, University of Catania, Catania, Italy

**Keywords:** pediatric and adolescent MEN 2, childhood medullary thyroid cancer, RET mutation, response to treatment, outcome, persistent disease, thyroid cancer

## Abstract

**Background:**

Multiple endocrine neoplasia type 2 syndrome (MEN2) is a hereditary disease resulting from mutations of the rearranged during transfection (RET) protooncogene subclassified into MEN2A [medullary thyroid carcinoma (MTC), pheochromocytoma, and primary hyperparathyroidism] and MEN2B (MTC, pheochromocytoma, Marfanoid habitus, mucous neuromas, and intestinal ganglioneuromatosis). Prophylactic thyroidectomy is recommended in RET-mutated patients. The age at which it should be performed depends on the type and aggressiveness of the mutation.

**Aim of the study:**

This study aimed to evaluate the genotype/phenotype correlation and outcome in pediatric/adolescent carriers of MEN2 RET mutation.

**Patients and methods:**

In a retrospective series of 23 carriers of RET MEN2 mutation who were ≤19 years old at diagnosis and had undergone total thyroidectomy ± lymphadenectomy, the following were analyzed: 1) specific RET mutation, 2) clinical and histopathological characteristics, 3) genotype/phenotype correlation, and 4) outcome at last follow-up.

**Results:**

In our series, the female gender was more prevalent (F/M ratio 2.8/1), and the median age was 14.9 years [interquartile range (IQR) 12.6–17.2]. RET mutations were at very high risk in 4.3% of patients (M918T), high risk in 43.5% (C634), and moderate risk in 52.2% (47.8% C618 and 4.3% C620). All patients underwent surgery: at histology, MTC was found in 19/23 (82.6%) patients, C-cell hyperplasia in 2/23 (8.7%), and benign histology in 2/23 (8.7%). Ten patients (52.6%) had a disease event during the follow-up: 2/19 (10.5%) showed biochemical disease, 6/19 (31.6%) lymph node recurrences, and 2/19 (10.5%) distant metastases (50% liver, 50% bone). At the last follow-up, nine MTCs were not cured. One patient died after 9 years of follow-up at 21 years old (M918T RET+).

**Conclusions:**

From these data, it is clear to see the importance of genetic counseling and RET screening in all first-degree relatives of patients with proven MEN2. The goal should be to subject patients to surgery for prophylactic and not curative purposes, i.e., before the onset of MTC, given the high risk of persistent or recurrent disease also in pediatric/adolescent patients.

## 1 Introduction

Medullary thyroid carcinoma (MTC) is a rare neoplasm (1%–2% of all thyroid tumors) that originates from the parafollicular cells of the thyroid. At diagnosis, all patients with MTC should be screened for rearranged during transfection (RET) mutations due to the fact that 20%–25% of cases have germline mutation being part of multiple endocrine neoplasia type 2 syndrome (MEN2) (1, 2).

MEN2 is a hereditary disease resulting from mutations of the RET protooncogene subclassified into MEN2A (MTC, pheochromocytoma, and primary hyperparathyroidism) and MEN2B (MTC, pheochromocytoma, Marfanoid habitus, mucous neuromas, and intestinal ganglioneuromatosis).

Hereditary MTC occurs at a younger age, is typically preceded by hyperplasia, and is often multifocal and/or bilateral (3). The age-related penetrance of hyperplasia and MTC depends on specific RET mutations.

Almost all patients with MEN2A (85%) are asymptomatic at diagnosis. The third decade is the age of the peak incidence in index patients.

Genetic counseling must be provided to all first-degree relatives of patients with proven MEN2.

At present, according to the American Thyroid Association (ATA) guidelines, patients are classified based on their phenotype (depending on the specific RET mutations) into three groups: highest, high, and moderate risk with progressive increases in aggressiveness (in terms of development at early age, often with local or distant metastases) (4–6). The ATA’s highest risk includes RET codon M918T mutation, high-risk RET codon C634 and A883F mutation, and moderate risk of all the other mutations.

The optimal timing of prophylactic thyroidectomy is recommended in RET-mutated patients depending on the aggressiveness of the mutation. Children in the highest ATA risk category should undergo total thyroidectomy within the first year of life, regardless of calcitonin level, which is naturally high in the first month of life. Children in the ATA high-risk category should undergo total thyroidectomy before the age of 5 or earlier based on their calcitonin values. Children in the ATA moderate-risk category generally develop a less aggressive tumor at an older age, so they should be screened every 6–12 months from the age of 5 and should undergo thyroidectomy in childhood or early adulthood, or sooner if elevated calcitonin values.

In some studies of children with MEN2 undergoing prophylactic surgery, no lymph node metastases or postoperative residual disease occurred when baseline serum calcitonin (Ctn) levels were <30–40 pg/mL (7–9).

A risk/benefit assessment between the risks of thyroidectomy and the possibility that MTC in patients with MEN2 will not be cured if thyroidectomy is delayed must be taken into account.

In the present study, we retrospectively evaluated a series of 23 pediatric/adolescent RET-mutated patients by analyzing clinical and histopathological characteristics, the specific RET mutation, the genotype/phenotype correlation, and the outcome at the last follow-up.

## 2 Patients and methods

A consecutive series of 23 patients with germline mutation of the RET protooncogene (MEN2), with an age at diagnosis ≤19 years, all followed up at Endocrinology Thyroid Clinic, Garibaldi-Nesima Medical Center, in Catania, from 1980 to 2018 were retrospectively analyzed. The median follow-up was 9.7 years, with a median of 4.1 and a range of 2–36 years. Two patients were index cases, and the other patients were identified during family screening.

Patients underwent total thyroidectomy (TT) ± lymphadenectomy (unilateral or bilateral) according to pre-surgery evaluation (mutation aggressiveness, ultrasound, and Ctn value). The criteria for lymph node (LN) central and/or lateral dissection were as follows: a) Ctn value >20 pg/mL (central) and >50 pg/mL (lateral), b) pre-surgery evidence of neck LN involvement either clinical or at ultrasound examination, and c) intra-surgery suspicion of metastatic LN.

The following data were collected: clinical (gender, age at diagnosis, and date of follow-up), histological (size of the primary tumor, multifocality, and number of metastatic lymph nodes at diagnosis), biochemical [Ctn and carcinoembryonic antigen (CEA) values at diagnosis and during follow-up], and morphological (computed tomography (CT), PET, magnetic resonance imaging (MRI), etc.) data and mutation status. Regarding genetic analysis, genomic DNA was purified from peripheral blood lymphocytes of the index case using the High Purification PCR Template Preparation Kit (Roche Diagnostic GmbH, Mannheim, Germany). RET gene exons 10, 11, 13, 14, 15, and 16 were analyzed using PCR and DNA direct sequencing. Exon 11 mutation was confirmed on an independent sample of the index case, and mutation analysis of his relatives was performed using specific amplification primers (5′-CCTCTGGCGGTGCCAAGCCTC-3′; 5′-CCTCGTCTGCCCAGCGTTG-3′) and exon 11 direct sequencing.

Tumors were staged according to the 8th TNM edition (10): T (primary tumor’s maximum size) and N (LN metastases) were assessed at pathological examination. The N status was indicated as N0 when all excised nodes were negative, N1a when positive nodes were only in the central compartment (levels VI–VII), and N1b when laterocervical nodes (levels I–V) were involved. M (distant metastases) was evaluated using total body imaging techniques [CT, MRI, whole-body bone scan (WBS), 18-fluorodeoxyglucose positron emission tomography (FDG-PET), and positron emission tomography with receptor tracers (for example, gallium, ^68^GA-PET)]. Patients were staged according to the TNM VIII edition.

All patients were periodically followed up by Ctn and CEA measurements, neck ultrasound, and further imaging methods when necessary. The intensity of subsequent examinations, every 6–12 months, was modulated on the basis of the initial risk assessment after surgery. At the last follow-up visit, persistence/recurrence of disease was defined by measurable values of Ctn and CEA or structural evidence of disease on ultrasound of the neck or other imaging methods. In particular, biochemical incomplete response is defined as detectable serum calcitonin and abnormal CEA with no evidence of structural disease. All patients who had persistent/relapsed disease during follow-up underwent additional diagnostic imaging procedures (CT, MRI, FDG-PET, ^68^GA-PET, and WBS), additional surgeries, and/or other therapies when required.

### 2.1 Statistical analysis

Categorical variables were expressed as frequencies and percentages. Normally distributed quantitative variables were expressed as mean ± standard deviation (SD), while non-normally distributed variables were expressed as median and interquartile range (IQR). The normality of quantitative variables was assessed using the Kolmogorov–Smirnov test. Categorical variables were analyzed using the chi-square test with Yates’s correction or Fisher’s exact test. A p-value <0.05 was considered statistically significant for all analyses. Data analysis was conducted using SPSS statistic software version 13.0 for Windows.

## 3 Results

### 3.1 Characteristics of the patients

The clinical and histopathological characteristics of the 23 pediatric/adolescent patients, six male and 17 female, carriers of RET MEN2 mutation, included in the present study are shown in **Table 1**.

**Table 1:** Clinical and histopathological characteristics of 23 RET-mutated patients at diagnosis.

	n.	%
Patients (n.)	23	
F/M	17/6	
Age (years)		
Median (IQR)	14.9 years (12.6–17.2)	
Surgery		
Total thyroidectomy (TT)	8	34.8
TT + central compartment dissection (CC)	11	47.8
TT + CC + omolateral laterocervical dissection	1	4.3
TT + CC + bilateral laterocervical dissection	3	13.0
T status		
T1a	14	60.9
T1b	1	4.3
T2	4	17.4
N status		
N0	11	47.8
N1a	4	17.4
N1b	2	8.7
Nx	2	8.7
Stage		
I	12	63.2
II	1	5.3
III	4	21.0
IV A	2	10.5
M status		
M1	2	8.7
Number of lymph node metastasis		
0 ≤5 N1 >5 N1	11/23 5/23 1/23	47.8 21.7 4.3
Bilaterality	11/23	47.8
Concomitant papillary thyroid cancer	1/23	4.3

RET, rearranged during transfection; IQR, interquartile range.

The differences in baseline characteristics between patients with high- and moderate-risk mutations are shown in **Supplementary Table S1**.

The median age at diagnosis was 14.9 years (IQR 12.6–17.2), and most patients were female (73.9%, F/M ratio 2.8/1). Total thyroidectomy was performed in eight patients (34.8%). LN surgery was performed in 15 (65.2%) patients: central compartment LN dissection in 11 and central and lateral LN dissection in four patients, of which three were bilateral. In 14 (60.9%), tumor size was ≤1 cm, and lymph node metastases were found in six (40.0%) patients who underwent lymph node dissection. At histology, MTC was found in 19/23 (82.6%) patients, C-cell hyperplasia in 2/23 (8.7%), and benign histology in 2/23 (8.7%). Two patients (8.7%) had distant metastasis at diagnosis (one liver and one bone).

Regarding the other manifestations of multiple endocrine neoplasia, only one patient was diagnosed with pheochromocytoma at 27 years old, and no patient developed hyperparathyroidism. The presence of the other manifestations of the syndrome in only a few cases is due to their young age and the later onset of pheochromocytoma or hyperparathyroidism.

**Figure 1:**
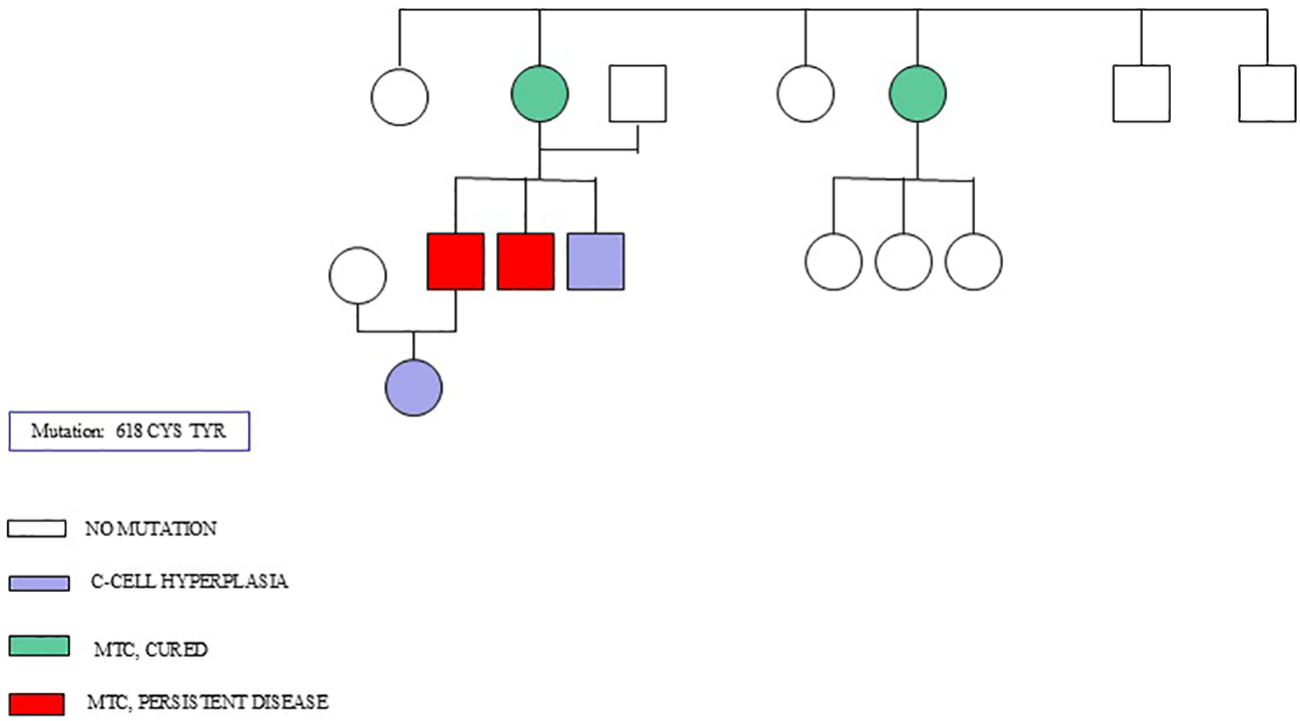
A genealogical tree of a family with 618 CYS TYR mutation.

**Figure 1** shows the genealogical tree of a family with a 618 CYS TYR mutation.

### 3.2 RET mutation

The mutations were at very high risk in 4.3% of patients (M918T), high risk in 43.5% (C634), and moderate risk in 52.2% (47.8% C618 and 4.3% C620).

Of patients with C618 mutation, 4/11 (36.4%) had C-cell hyperplasia/negative histology; instead, in all patients with other mutations, MTC was already diagnosed.

### 3.3 Disease events and response to therapy at last follow-up

Ten of 19 (52.6%) had a disease event related to MTC during the follow-up: 2/19 (10.5%) biochemical disease, 6/19 (31.6%) lymph node recurrences, and 2/19 (10.5%) distant metastases (50% liver and 50% bone).

During follow-up, due to disease persistence, 5/6 patients (26.3%) underwent reoperation with subsequent histological diagnosis of lymph node metastasis.

At the last follow-up, nine MTCs were not cured (55.6%% with RET C634 mutation, 33.3% with C618, and 11.1% with M918T). All patients had positive calcitonin values and lymph node metastases, and two patients had distant metastases. Analyzing the rate of persistent disease according to the mutation, 5/10 (50%) had RET C634 mutation, 3/11 (27.3%) had C618, and 1/1 (100%) had M918T.

We analyzed the outcome in the two main groups of patients according to the mutation (618 vs. 634 mutated patients), and there was no significant difference (p = 0.38), probably due to the sample size.

One patient died after 9 years of follow-up at 21 years old (M918T). Among N1 patients, the number of lymph node metastases ranged from 1 to 8 (median 3). Most N1 patients 83.3% (5/6) were not cured at the last follow-up.

### 3.4 Outcome at last follow-up according to RET mutation and the age of carrier relatives

We evaluated whether the carrier’s age at diagnosis could have influenced the late diagnosis and therefore the surgery in our patients, resulting in a high percentage of medullary carcinoma diagnoses and disease persistence (**Table 2**).

**Table 2:** Outcome at last follow-up according to RET mutation and the age of carrier relatives.

Pts	RET mutation and MTC ATA risk	Age carrier	Age at thyroidectomy	Recommended age (ATA guidelines(2))	TNM	Stage	Outcome (last control)	Follow-up (years)
1	618 (moderate)	68	10	According to CT and US	Benign		–	7.3
2	618 (moderate)	30	15	According to CT and US	Benign		–	6.3
3	618 (moderate)	68	19	According to CT and US	T1amN0Mx	I	Disease-free	8
4	M918T (Highest)	Case index	12	<1 year	T2N1bM1	IVA	Disease	9.1
5	620 (moderate)	34	14	According to CT and US	T1aN0Mx	I	Disease-free	9.7
6	618 (moderate)	66	14	According to CT and US	Benign		–	6.3
7	634 (high)	53	17	<5 years	T2mN0Mx	II	Disease	28.4
8	634 (high)	52	5	<5 years	T1aN0Mx	I	Disease-free	10.6
9	634 (high)	52	9	<5 years	T1aN0Mx	I	Disease-free	11.4
10	634 (high)	Not known	17	<5 years	T2N1aM0	III	Disease	7.6
11	618 (moderate)	66	16	According to CT and US	T1aN0Mx	I	Disease-free	5.4
12	618 (moderate)	66	12	According to CT and US	T1aNxMx	I	Disease	5.2
13	618 (moderate)	66	12	According to CT and US	T1aN0Mx	I	Disease-free	2.6
14	618 (moderate)	66	16	According to CT and US	T1amN0Mx	I	Disease	11.2
15	618 (moderate)	66	4	According to CT and US	Benign		–	12.6
16	618 (moderate)	38	14	According to CT and US	T1amN1aMx	III	Disease	15.7
17	618 (moderate)	38	11	According to CT and US	T1am N0Mx	I	Disease-free	14.4
18	634 (high)	16	16	<5 years	T1aNxMx	I	Disease-free	12.3
19	634 (high)	16	16	<5 years	T1N1bM1	IVA	Disease	37.2
20	634 (high)	16	17	<5 years	T2N1aMx	III	Disease	15.6
21	634 (high)	44	14	<5 years	T1aN1aMx	III	Disease-free	5.8
22	634 (high)	44	8	<5 years	T1aN0Mx	I	Disease-free	4.1
23	634 (high)	Case index	19	<5 years	T1aN0Mx	I	Disease	6.1

Pts, patients; RET, rearranged during transfection; MTC, medullary thyroid carcinoma; ATA, American Thyroid Association.

All four patients with benign histology were thyroidectomized at a young age (4, 10, 14, and 15 years old) and had a moderate risk of RET mutation.

Evaluating the median age at thyroidectomy and the outcome (disease or disease-free) according to the RET mutation risk, we found the following:

1. no difference in children with moderate risk RET mutations (median, 14 years old in both groups, p = 1), and
2. significant difference in children with high-risk RET mutations (disease-free 9 years old and 17 years old in the other group, p = 0.04).

## 4 Discussion

MTC is a rare tumor accounting for approximately 1–2% of all thyroid tumors. Approximately 20%–25% of cases have germline mutation of RET being included in MEN2, so at diagnosis, genetic counseling and RET screening must be provided to all first-degree relatives of patients with proven MEN2.

Hereditary MTC is usually preceded by hyperplasia. The age of onset of hyperplasia and MTC depends on the specific RET mutation.

At present, three groups of risk, depending on the specific RET mutations, have been identified (2): highest (including RET codon M918T mutation), high (including RET codon C634 and A883F mutations), and moderate risk (including all the others RET mutations) with progressive increases in aggressiveness in terms of onset and the stage, local, or distant disease (4, 6).

The optimal timing of the thyroid screening (with ultrasound and calcitonin determination) and the prophylactic thyroidectomy is recommended in RET-mutated patients according to the risk category. For the highest-risk category, total thyroidectomy is recommended within the first year. For the high-risk category, it is recommended that patients undergo a physical examination, serum calcitonin level test, and neck ultrasound from the age of 3 years and total thyroidectomy before 5 years old. For the moderate-risk category generally, patients should be screened every 6–12 months from the age of 5 and undergo surgery in childhood/early adulthood (earlier if calcitonin value is elevated) (10, 11).

The goal in relatives of patients having MEN2 syndromes is the presymptomatic detection of the RET mutation and consequently the correct timing of follow-up and/or thyroidectomy before the onset of MTC (12–16).

The hereditary form of MTC, being part of MEN2 syndrome, is subclassified into two forms: MEN2A (95%) and MEN2B (5%). In our series, only one patient (4.3%) had M918T RET mutation (MEN2B), similar to literature data.

MEN2A is characterized by the presence of MTC associated with pheochromocytoma hyperparathyroidism or both, with a different frequency depending on the specific RET mutation.

In our series, only one patient had the diagnosis of pheochromocytoma at 27 years old, and no patient developed hyperparathyroidism; instead, in their relatives, seven developed pheochromocytoma and two hyperparathyroidism. Therefore, the presence of the other manifestations of the syndrome in only a few cases is due to their young age and the later onset of pheochromocytoma or hyperparathyroidism.

The median age in our series was 14.9 years, older than 10 years reported in a paper by Sanso et al., which included 60 patients aged 6 months to 21 years, with 18 thyroidectomized (17).

Histologically, 21/23 (91.3%) patients had parafollicular cell hyperplasia, 19/23 (82.6%) had MTC, and 2/23 (8.7%) had benign histology. These data are similar to those shown in the paper of Sanso (17): 18/18 (100%) had parafollicular cell hyperplasia and 15/18 (83.3%) had MTC.

Regarding lymph node metastases in our series, 6/19 (31.6%) had N1a or N1b involvement, higher than in the Sanso series (3/15, 20%).

Of patients with C618 mutation, 4/11 (36.4%) had C-cell hyperplasia/negative histology; instead, in all patients with other mutations, MTC was already diagnosed.

Our data are different from the data reported in the literature by Romei et al. (66), who found a significantly higher frequency for the Val804met mutation of the non-cysteine region in exon 14 of the RET gene.

In a recent paper by Elisei et al. (18), 2,031 patients (1,264 with sporadic MTC, 117 with hereditary MTC, and 650 relatives) were evaluated. They found that in the group of clinically familial cases, Cys634 mutations were the most prevalent (31.6%), following the V804M RET mutation (19.6%).

In our series, 1/23 patients (4.3%) presented an association between MTC and papillary/follicular thyroid carcinoma. The coexistence of MTC and papillary/follicular thyroid carcinoma is a rare phenomenon that occurs in a range of 1% to 19% of all thyroid tumors in relation to the different series and must be considered incidental, as one cannot, however, exclude the possibility of a shared pathogenetic mechanism of both tumors (19, 20). Progression and prognosis are similar to the predominant component of the tumor. Radioactive iodine therapy can be used for follicular components.

As reported by several authors, the presence of lymph node metastases, the localization of N1, and the number of lymph nodes involved at diagnosis represent negative prognostic factors in patients with MTC (21, 22).

After the initial surgical treatment, in our series, the disease at the last follow-up was 39%, 23.5% in N0 patients, and 83.3% in N1 patients (75% in N1a and 100% in N1b).

During the follow-up, 10/19 patients (52.6%) had a disease event: 10.5% biochemical disease, 31.6% lymph node recurrences, and 10.5% distant metastases (liver and bone). During follow-up, due to disease persistence, five (26.3%) underwent reoperation with a histological diagnosis of lymph node metastasis.

At the last follow-up, nine MTCs were not cured. All patients had positive calcitonin values and lymph node metastases, and two patients had distant metastases. Analyzing the rate of persistent disease according to the mutation, 5/10 (50%) had RET C634 mutation, 3/11 (27.3%) C618, and 1/1 (100%) M918T. The only patient who died after 9 years of follow-up at 21 years old had M918T, confirming the literature data of the greater aggressiveness of MEN2B.

The limitations of the present analysis were that it is a retrospective study with possible enrollment bias and that the total number of patients was not numerous, depending on both the fact that it was a single-center study and the rarity of the syndrome.

## 5 Conclusions

From these data, it is clear to see the importance of genetic counseling and RET screening in all first-degree relatives of patients with proven MEN2. The objective should be to identify the correct timing of screening (calcitonin and ultrasound) and thyroidectomy before the onset of MTC develops or while it is clinically not evident and confined to the gland.

It is clear that the late diagnosis of MTC, understood as an advanced stage of the disease (stages III–IV), is associated with a high risk of persistent or recurrent disease also in pediatric/adolescent patients.

## Data Availability

The data supporting the findings of this study are derived from clinical records and known RET proto-oncogene mutations, which are publicly available in databases such as ClinVar. Due to ethical and privacy constraints, individual patient data cannot be shared publicly. De-identified data may be made available upon reasonable request to the corresponding author, in compliance with applicable legal and ethical standards.

## Supplementary material

Supplementary Table 1 Differences in baseline characteristics between patients with high and moderate risk mutations.
