# Supplementary material for: Medullary thyroid cancer in MEN2 pediatric/adolescent carriers of RET mutation: genotype/phenotype correlation and outcome in a retrospective series of 23 patients
Source: Front Oncol. 2025 Jan 7;14:1464890. doi: 10.3389/fonc.2024.1464890 (PMC11746086; doi:10.3389/fonc.2024.1464890)
Supplement: Supplementary Table 1 — Differences in baseline characteristics between patients with high and moderate risk mutations. [file Table1.docx]

Supplementary table 1 Differences in baseline characteristics between patients with high and moderate risk mutations.

|  | | | n. | % | n. | % |
| --- | --- | --- | --- | --- | --- | --- |
| RET mutation | | | High Risk | | Moderate risk | |
| Patients (n.) | | | 11 |  | 8 |  |
| F/M | | | 9/2 |  | 5/3 |  |
|  | Age (years) | |  | |  |  |
|  |  | median (IQR) | 16.4 (10.8-17.6) | | 14.6 (12.9-16.2) | |
|  | Surgery | |  | |  |  |
|  |  | Total thyroidectomy (TT) | 2 | 18.2 | 2 | 25 |
|  |  | TT + central compartment dissection (CC) | 5 | 45.5 | 6 | 75 |
|  |  | TT+CC+ omolateral laterocervical dissection | 1 | 9.1 | 0 |  |
|  |  | TT+CC+ bilateral laterocervical dissection | 3 | 27.3 | 0 |  |
|  | T status | |  |  |  |  |
|  |  | T1a | 6 | 54.5 | 8 | 100 |
|  |  | T1b | 1 | 9.1 | 0 |  |
|  |  | T2 | 4 | 39.4 | 0 |  |
|  | N status | |  | |  |  |
|  |  | N0 | 5 | 45.5 | 6 | 75 |
|  |  | N1a | 3 | 27.3 | 1 | 12.5 |
|  |  | N1b | 2 | 18.2 | 0 |  |
|  |  | Nx | 1 | 9.1 | 1 | 12.5 |
|  | Stage | |  |  |  |  |
|  |  | I | 5 | 45.5 | 7 | 87.5 |
|  |  | II | 1 | 9.1 | 0 |  |
|  |  | III | 3 | 27.3 | 1 | 12.5 |
|  |  | IV A | 2 | 18.2 | 0 |  |
|  | M status | |  |  |  |  |
|  |  | M1 | 2 | 18.2 | 0 |  |
|  | **Number of lymph node metastasis**  - 0  - ≤5 N1  - >5 N1 | | 4  4  1 |  | 5  1  0 |  |
|  | Bilaterality | | 7 | 63.6 | 4 | 50 |
|  | Concomitant papillary thyroid cancer | | 1 | 9.1 | 0 |  |
